# Region‐specific increase in CK2 expression is associated with decreased 3R:4R tau ratio in AD patients

**DOI:** 10.1002/alz70855_104015

**Published:** 2025-12-24

**Authors:** Simone Graziano, Lila Shapiro, Phoebe Calero, Courtney Marshall

**Affiliations:** ^1^ Wellesley College, Wellesley, MA, USA

## Abstract

**Background:**

Tauopathies can be categorized by the type of tau isoforms that comprise pathological aggregates: 3R, 4R, or 3R and 4R. We previously demonstrated an increase in Casein Kinase 2 (CK2), which positively correlated with hyperphosphorylated tau in the hippocampus of postmortem Alzheimer's disease (AD) patients. These findings were singular to AD and not observed in single‐isoform tauopathies, such as Corticobasal Degeneration, Progressive Supranuclear Palsy, or Pick's disease. We investigated whether this unique result is related to the AD‐specific pathology of mixed 3R/4R tau isoforms.

**Method:**

We immunohistochemically investigated the regional expression of CK2 in postmortem samples from AD patients (*n* = 13) and age‐matched individuals (*n* = 9) with primary age‐related tauopathy (PART) within the thalamus, amygdala, and visual cortex. The 3R:4R tau isoform ratio was analyzed within the hippocampus, thalamus, amygdala, and visual cortex of AD patients.

**Result:**

We found that hippocampal CK2 in AD patients positively correlates with the ratio of 3R:4R tau, but does not significantly relate to the expression of either isoform individually. In addition, the ratio of 3R:4R tau in AD patients is skewed in the hippocampus compared to other brain regions. Specifically, the hippocampus has significantly more 4R tau than 3R tau, while the thalamus, amygdala, and visual cortex have an approximate 1:1 ratio. Compared to age‐matched controls, we did not observe increased CK2 expression within the three brain regions containing balanced ratios of tau isoforms.

**Conclusion:**

This data suggests that the previously identified positive correlation between CK2 and hyperphosphorylated tau in AD patients may be connected to the unique pathogenesis of a mixed‐isoform tauopathy. Aberrant hippocampal CK2 expression may influence region‐specific increases in 4R tau relative to 3R tau and drive other AD‐specific aspects of pathology. These findings support future research involving the inhibition of CK2 as a potential therapeutic intervention in AD.

